# Distribution of hepatitis B virus-positive individuals in Zaria, Nigeria, according to risk-associated practices

**DOI:** 10.25259/cjhs_7_2019

**Published:** 2020-02-04

**Authors:** Abdurrahman El-Fulaty Ahmad, Adamu Girei Bakari, Bolanle O. P. Musa, Shettima K. Mustapha, Idris Nasir Abdullahi, Mohammed Ibrahim Tahir, Bello Yusuf Jamoh, Abdulqadri O. Olatunji, Sumayya Hamza Maishanu, Bello Hali, Claudia A. Hawkins, Atiene S. Sagay, Adebola T. Olayinka

**Affiliations:** 1 Department of Medical Laboratory Science, Ahmadu Bello University, Zaria,; 2 Department of Medicine, Ahmadu Bello University, Zaria,; 3 Department of Research Unit, DNA LABS, Unguwar Sarki, Kaduna,; 4 Department of Microbiology, Infectious Diseases Laboratory, Usmanu Danfodiyo University Teaching Hospital, Sokoto,; 5 Department of Medicine, Division of Infectious Diseases, Feinberg School of Medicine, Northwestern University, Chicago, Illinois, United States.; 6 Department of Obstetrics and Gynaecology, University of Jos, Jos, Nigeria,; 7 Department of Medical Microbiology, Ahmadu Bello University, Zaria,

**Keywords:** Hepatitis B virus, Risks, Perceptions, Social media, Zaria

## Abstract

**Background:**

An estimated 75% of Nigerians are at risk of hepatitis B virus (HBV) exposure. In an attempt to reduce the menace, the assessment of risk factors associated with HBV infection and general perception of infected individuals is a step in that direction.

**Aim of the Study:**

This study, therefore, identified exposure to risk factors and general perceptions associated with HBV infection in infected individuals in Zaria, Nigeria.

**Methodology:**

Four milliliters of blood were collected in ethylenediaminetetraacetic acid container from each of 165 HBV surface antigen (HBsAg)-positive participants recruited purposively from the gastroenterology clinic of ABUTH Zaria from May to August 2017. Plasma was separated and used to screen for HBsAg with Fastep® rapid strip. Epi Info® questionnaire database was used to collate data on sociodemographics, risk factors, and perception indices. GraphPad Prism 6 was used for statistical analysis.

**Results:**

The median interquartile range age of the participants was 31.0 (25.5–39.0) years with 107 (64.8%) male participants. Sharing hair clippers, commercial pedicure, and body piercing among others were some of the risks that the study participants reported to be exposed to. One-quarter of health workers involved in the study had needlestick injury. Less than half of the study participants (47.7%) knew of hepatitis B before testing HBsAg seropositive. Knowledge of the HBV vaccine before testing and adherence was generally poor (38.6% and 44.6%, respectively). There was a significant linear relationship between the level of education and knowledge of hepatitis B.

**Conclusion:**

Considering the myriads of already established risks of HBV seen in Zaria, massive enlightenment campaigns need to be embarked on continuously through all available media, including social media.

## INTRODUCTION

Hepatitis B virus (HBV) is one of the major causes of morbidity and mortality worldwide.^[1]^ About 2 billion people are thought to have evidence of past or present infection with HBV, with about 240 million chronic carriers of HBV surface antigen (HBsAg).^[2]^ Worldwide, approximately 650,000 people die each year from complications of chronic hepatitis B.^[3]^ In Nigeria, HBV infection is hyperendemic with the seroprevalence of HBsAg ranging from 10% to 40%.^[4–8]^ About 75% of the Nigerian adult population is at risk of exposure to the virus infection translating to a huge risk of contracting HBV infection.^[9]^

Factors associated with acquiring HBV infection include needlestick injuries, sharing hair clippers and pedicure tools, dental procedures, and cultural practices such as tribal marks, traditional circumcision, and tattoo inscriptions. HBV carrier mothers and HBV-positive sexual partners are potential sources of HBV transmission. Immunization conferred by the HBV vaccine regimens reduces the burden of HBV and its contending risks.^[8]^

Assessment of such risk factors associated with HBV infection and the perception of infected individuals in this environment may help in identifying strategies for educating HBV infection-prone population on ways and manners to reduce the menace. This study, therefore, identified risk factors and general perceptions associated with HBV infection in infected individuals in Zaria, Nigeria.

## MATERIALS AND METHODS

This study was conducted in the gastroenterology clinic of the Ahmadu Bello University Teaching Hospital, Shika, Zaria, Nigeria, from May to August 2017. One hundred and sixty-five study participants with known HBV infection were recruited consecutively and 4 mL of their venous blood samples collected in ethylenediaminetetraacetic acid (EDTA) container. The sample size was deduced from a national prevalence rate of 12.2%.^[8]^ The EDTA-anticoagulated blood was centrifuged at 5000 rpm for 5 min to separate the plasma. The clear layer of plasma was then transferred into cryovials and stored at −20°C till analysis. Fastep® rapid immunochromatographic strip kit was used for the analysis and it was brought to room temperature before use. The test kit was removed from its sealed pouch and placed on a clean level surface. The kit was labeled with the participant’s identification number. Using a Pasteur pipette, two drops of plasma and one drop of buffer were added to the sample pad of the strip. The timer was set for 10 min. The results were read before 20 min.

Data concerning sociodemographic characteristics, exposures to risk factors, and perception indices were collated and validated using Epi Info® questionnaire database. Structured questionnaires were administered to the participants at the point of recruitment with 100% retrieval. Univariate analysis of the sociodemographic characteristics and risk factors for HBV infection was conducted. Chi-square test for trend was used to determine the relationship between the level of education of participants and their knowledge of HBV infection risk factors, while Chi-square test with Yates correction was used to determine the relationship between their knowledge of HBV and occupation. Statistical analysis was conducted with GraphPad Prism 6 statistical software package.

Ethical approval was obtained from the Health Research Ethics Committee of the Ahmadu Bello University Teaching Hospital, Zaria, before the commencement of sample collection. Written informed consent was sought and obtained from each participant before enrollment into the study as all participants were adults. In the informed consent forms, the participants were properly informed of their rights. All data were treated with utmost confidentiality.

## RESULTS

### Sociodemographic characteristics of HBsAg-positive study participants in Zaria, Nigeria

One hundred and sixty-five HBsAg-positive participants were recruited for the study with a median (and interquartile range) age of 31.0 (25.5–39.0) years. Male participants constituted 64.8% (107/165) of the total number recruited. A decreasing trend of frequency with increase in age was observed such that the age group of 18–27 years constituted the highest number of participants with 62 (37.6%) while the age group of 48–57 years had a frequency of 15 (9.1%). Majority of the participants were with a form of education stratified at different levels with only 1 (0.6%) individual without any form of education. Civil servants had the highest number of participants with 49 (29.7%) with only 1 (0.6%) retiree, being the least. Ninety-nine (60%) of the study participants were married men and women while 3 (1.8%) of the participants were divorced [Table 1].

### Exposure to factors associated with the risk of HBV infection among participants

Seventy-five (70%) of the male participants had traditional circumcision. Other risk factors that the study participants were exposed to included sharing hair clippers, drips administration, commercial pedicure, dental procedure, body piercing and tribal marks with frequencies of 100 (60.6%), 105 (63.6%), 65 (39.4%), 57 (34.5%), 54 (32.7%) and 53 (32.1%), respectively. Of the health workers who participated in the study, in their response to whether they had needlestick injury, 8 (25%) answered in affirmative. Other responses relate to risks of hepatitis B infection among the study participants, which had relatively lower figures included women delivering at home, parents/spouses with hepatitis B, local practices of surgery, and coming in contact with blood; 13 (22.4%), 27 (16.4%), 23 (13.9%), and 17 (10.3%), respectively. Twenty-two (13.3%) participants had blood transfusion and hospital surgeries each, while tattoo inscriptions were observed on only 3 (1.8%) of the participants [Table 2].

### General perception of the study participants with regard to HBV infections and associated risks

Of the 99 married participants that responded, 67 (67.7%) were in monogamous marriage, while the remaining 32 (32.3%) were in polygamous marriages. Of the 108 participants that had sexual partners, 80 (74.1%) had only one sexual partner each, while each of 17 (15.7%), 7 (6.5%), 3 (2.8%), and 1 (0.9%) had two, three, four, and more than four sexual partners, respectively. Among the 107 participants that responded to the question as to whether they informed their sexual partners of their hepatitis B status, 94 (87.9%) claimed to have informed their partners of their hepatitis B status. Among the 94 who informed their partners of their status, 21 (30.0%) of their partners tested positive for HBV, while 24 (34.3%) did not get their partners to be tested [Table 3].

Of the total study participants, a significant figure of 153 (92.7%) had heard of hepatitis B, while 12 (7.3%) had no prior knowledge of HBV despite having tested positive for the virus. Out of those that had heard of hepatitis B, 64 (47.7%) only knew about it after they tested positive [Table 3].

Moreover, out of the total participants, 101 (61.2%) knew of the availability of hepatitis B vaccine, among whom only 39 (38.6%) knew before they tested for HBsAg seropositivity [Table 3].

Only 15 (9.1%) of the total participants had received the HBV vaccine. Of these, only 2 (13.3%) received all three dosages of the vaccine and along with booster dose(s), 5 (33.3%) received exactly three dosages, while all the rest had incomplete dosages [Table 3].

There was a significant linear relationship (*χ*^2^ test for linear trend: 6.870, df = 1, *P* = 0.0088) between the levels of education of the study participants and their knowledge of hepatitis B [Table 4].

Of the 49 study participants that were civil servants, 48 (98.0%) knew about hepatitis B, while 1 (2.0%) did not know [Table 5]. Of the 40 participants that were self-employed, 37 (92.5%) knew of the disease and 3 (7.5%) did not know. All the participants were non-governmental employees (*n* = 3) and the only retiree was enrolled knew of hepatitis B. Of the 38 students that were enrolled in the study, 36 (94.7%) knew of hepatitis B, while 2 (5.3%) did not. Twenty-five (83.3%) of 30 housewives that participated in this study knew about hepatitis B and 5 (16.7%) did not. The remaining four participants with other forms of occupation comprised 3 (75.0%) individuals that knew about hepatitis B and 1 (25.0%) who did not. Statistical analysis showed no relationship between the occupation of the study participants and their knowledge of hepatitis B (*χ*^2^ test for independence: 8.323, df = 6, *P* = 0.2154) [Table 5].

## DISCUSSION

### Risk factors associated with HBV infection

This study noted some common practices that are established risk factors for HBV infection such as sharing barbers hair clippers, having tribal marks, living with HBV-infected partners, traditional surgeries and circumcisions, body/ear piercing in females, and commercial pedicure, all of which are common social practices in the study area. Dental procedures, child delivery at home with untrained midwives attending, and needlestick injuries among health workers were also already established risk factors for HBV infection observed in Zaria, which might have an effect on transferring HBV.^[8,10,11]^

### General perception toward HBV

This study also noted that most of the participants were in monogamous marriage. Furthermore, most of the participants were having only one sexual partner. This might suggest minimal risk of sexually transmitted HBV infection. It was also noted that most of them informed their sexual partners of their hepatitis B status. Among the majority of the partners tested for HBV, more than half were negative. This is a clear indication that in this study area, HBV infection is not considered a stigmatizing infection like that of HIV.

We observed that most of the study participants had knowledge of hepatitis B infection with about half of them having the awareness after they had tested positive for the virus. Even while attending the gastroenterology clinic due to HBV infection, some participants did not have any knowledge of hepatitis B. This is suggestive of the fact that a large portion of the general population was not getting voluntary testing for HBV, which could partly be due to lack of any knowledge of the virus. A significant number of HBV-infected people were first diagnosed with the virus at the blood donation bays.^[12]^ Second, a strong association was noted between the level of education of the participants and their knowledge of hepatitis B. This finding agrees with those in Korea which could be corroborated by the fact that the more knowledgeable one is, the more likely he or she is to know about general health issues which are directly or indirectly, gotten from academic gatherings such as seminars, health talks, posters, and stickers.^[13,14]^ More so, the enlightened ones are usually the first target for health talks and awareness campaigns. Massive enlightenment campaigns need to be embarked on continuously through all available media such as social media, media houses, and roadside posters to extend the message to the less and uneducated persons.

Moreover, most of the participants knew about the availability of the HBV vaccine after testing positive. A large majority of the participants had no prior history of receiving HBV vaccines indicating their susceptibility to HBV infection. The few that were vaccinated were either never immunized or received the vaccine while already infected with HBV, as evidently majority did not have complete regimens. This calls for adherence to full vaccination regimens for susceptible persons and encourages assaying for hepatitis B surface antibody quantification to ascertain immunization.

This study, however, did not assess responses to the attitude of participants, particularly health workers with needlestick injury and use of personal or commercial sharp objectives at barbers and pedicure shops. It also did not assess how the participants knew of their infection with HBV. This is open to further investigations.

## CONCLUSION

Considering the myriads of already established risks of HBV seen in Zaria, massive enlightenment campaigns need to be embarked on continuously through all available media, including social media.

## Figures and Tables

**Table 1:** Sociodemographic characteristics of hepatitis B virus surface antigen-positive study participants in Zaria, Nigeria (*n*=165).

Median (interquartile range) age: 31.0 (25.5–39.0) years		
Variable	Frequency (*n*=165)	% (95% CI)
Sex		
Male	107	64.8 (57.0–72.1)
Female	58	35.2 (27.9–43.0)
Age group (years)		
18–27	62	37.6 (30.2–45.4)
28–37	55	33.3 (26.2–41.1)
38–47	33	20.0 (14.2–26.9)
48–57	15	9.1 (5.2–14.6)
Level of education		
Primary	9	5.5 (2.5–10.1)
Secondary	46	27.9 (21.2–35.4)
Tertiary	80	48.5 (40.6–56.4)
Postgraduate	12	7.3 (3.8–12.4)
Qur’anic/Islamiyya only	17	10.3 (6.1–16.0)
None	1	0.6 (0.01–3.3)
Occupation		
Civil servant	49	29.7 (22.8–37.3)
Self-employed	40	24.2 (17.9–31.5)
Non-governmental employee	3	1.8 (0.4–5.2)
Retired	1	0.6 (0.02–3.3)
Student	38	23.0 (16.8–30.2)
Housewife	30	18.2 (12.6–24.9)
Others	4	2.4 (0.7–6.1)
Marital status		
Single	59	35.8 (28.5–43.6)
Married	99	60.0 (52.1–67.5)
Divorced	3	1.8 (0.4–5.2)
Widowed	4	2.4 (0.7–6.1)

Univariate analysis showing the frequency and percentages with 95% CI. CI: Confidence interval

**Table 2:** Factors associated with the risk of hepatitis B infection among the study participants (*n*=165).

Risk factor	Frequency	% (95% CI)
Tattoo	3	1.8 (0.4–5.2)
Tribal marks	53	32.1 (25.1–39.8)
Parents/partner (s) with hepatitis B	27	16.4 (11.1–22.9)
Sharing hair clipper	100	60.6 (52.7–68.1)
Needlestick injury (*n*=32)*	8	25.0 (11.5–43.4)
Child delivery at home (*n*=58)*	13	22.4 (12.5–35.3)
Orthodox surgery	22	13.3 (8.5–19.5)
Traditional surgery	23	13.9 (9.0–20.2)
Traditional circumcision (*n*=107)*	75	70.1 (60.6–78.6)
Blood transfusion	22	13.3 (8.5–19.5)
Drip	105	63.6 (55.8–71.0)
Dental procedure	57	34.5 (27.3–42.3)
Body piercing	54	32.7 (25.6–40.5)
Contact with blood	17	10.3 (6.1–16.0)
Commercial pedicure	65	39.4 (31.9–47.3)

* Not applied to all respondents. Univariate analysis showing the frequency and percentages with 95% CI. CI: Confidence interval

**Table 3:** General perceptions of the study participants with regard to hepatitis B virus infections and associated risks.

Variable	Frequency	% (95% CI)
Nature of marriage (*n*=99)		
Monogamy	67	67.7 (57.5–76.7)
Polygamy	32	32.3 (23.3–42.5)
Number of sexual partners (*n*=108)		
1	80	74.1 (64.8–82.0)
2	17	15.7 (9.4–24.0)
3	7	6.5 (2.6–12.9)
4	3	2.8 (0.6–7.9)
>4	1	0.9 (0.02–5.1)
Informed partner (s) of hepatitis B status (*n*=107)		
Yes	94	87.9 (80.1–93.4)
No	13	12.1 (6.6–19.9)
If yes, were they tested? (*n*=94)		
Yes	70	74.5 (64.4–82.9)
No	24	25.5 (17.1–35.6)
If tested, results (*n*=70)		
Positive	21	30.0 (19.6–42.1)
Negative	48	68.6 (56.4–79.1)
Do not know	1	1.4 (0.04–7.7)
Ever heard of hepatitis B (*n*=165)		
Yes	153	92.7 (87.6–96.2)
No	12	7.3 (3.8–12.4)
If yes, time (*n*=153)		
Before testing	80	52.3 (44.1–60.4)
After testing	73	47.7 (39.6–55.9)
Ever heard of hepatitis B vaccine? (*n*=165)		
Yes	101	61.2 (53.3–68.7)
No	64	38.8 (31.3–46.7)
If yes, time (*n*=101)		
Before testing	39	38.6 (29.1–48.8)
After testing	62	61.4 (51.2–70.9)
Ever received the hepatitis B virus vaccine (*n*=165)		
Yes	15	9.1 (5.2–14.6)
No	150	90.9 (85.4–94.8)
If yes, number of shots received (*n*=15)		
1	2	13.3 (1.7–40.5)
2	6	40.0 (16.3–67.7)
3	5	33.3 (11.8–61.6)
>3	2	13.3 (1.7–40.5)

Univariate analysis showing the frequency and percentages with 95% CI. CI: Confidence interval

**Table 4:** Relationship between the level of education of the participants and their knowledge of hepatitis B.

Education level	Heard of hepatitis B (%)		Total (%)
	Yes	No	
Primary	7 (4.7)	2 (1.4)	9 (6.1)
Secondary	42 (28.6)	4 (2.7)	46 (31.3)
Tertiary	78 (53.1)	2 (1.4)	80 (54.4)
Postgraduate	12 (8.2)	0 (0.0)	12 (8.2)
Total	139 (94.6)	8 (5.4)	147 (100)

*χ*^2^ test for independence: 8.323, df=6, *P=*0.2154

**Table 5:** Relationship between occupations of the study participants and their knowledge of hepatitis B.

Occupation	Awareness of hepatitis B (%)		Total (%)
	Yes	No	
Civil servant	48 (98.0)	1 (2.0)	49 (100.0)
Self-employed	37 (92.5)	3 (7.5)	40 (100.0)
Non-governmental employee	3 (100.0)	0 (0.0)	3 (100.0)
Retired	1 (100.0)	0 (0.0)	1 (100.0)
Student	36 (94.7)	2 (5.3)	38 (100.0)
Housewife	25 (83.3)	5 (16.7)	30 (100.0)
Others	3 (75.0)	1 (25.0)	4 (100.0)
Total	153 (92.7)	12 (7.3)	165 (100.0)

*χ*^2^ test for independence: 8.323, df=6, *P=*0.2154
